# Impact of different forms of selenium supplementation on growth and physiological performance of New Zealand white rabbits

**DOI:** 10.1007/s11250-024-03970-8

**Published:** 2024-04-19

**Authors:** Kout-Elkloub M. El. Moustafa, Hoda M. EL-Hosseiny, G. F. Shaheen, E. M. El-Kotamy, Abd elghani Ghoniem, G. E. Younan, M. M. El-Nahrawy, Mona E. Farag, Manal S. Mohamed

**Affiliations:** 1 Department of Poultry Nutrition Research, Animal Production Research Institute (APRI), Agricultural Research Center (ARC), Dokki, Giza, Egypt; 2 Department of Animal Nutrition Research, Animal Production Research Institute (APRI), Agricultural Research Center (ARC), Dokki, Giza, Egypt; 3 Department of Rabbits, Turkey and Waterfowl Breeding Research, Animal Production Research Institute (APRI), Agricultural Research Center (ARC), Dokki, Giza, Egypt

**Keywords:** Rabbits, Nano-Se, Productive performance and Economic efficiency

## Abstract

Forty-eight weaned male New Zealand White rabbits aged 6 weeks with an initial body weight of (709.67 ± 13 g) were randomly divided into six experimental groups (8 rabbits each) for 6–14 weeks of age experimental periods. The present study was planned to evaluate the effect of using different forms of Selenium element (inorganic, nanoparticles and organic) as dietary supplementation on productive performance of rabbits. Six experimental groups in completely randomized design were used. The first group (G_1_, control) was fed the basal diet to cover maintenance and production allowances. Rabbits in the other groups G_2_, G_3_, G_4_ and G_5_ were fed the basal diet supplemented with Nano- Se at 0.02, 0.03, 0.04 and 0.05 mg/kg diet, respectively. The 6th group (G_6_) was fed the basal diet supplemented with 0.1 mg/kg diet of salinized yeast (Se-yeast) as organic form. The results indicated that the highest values of nitrogen free extract (NFE) and crude fiber (CF) digestibility, live body weight, daily weight gain, hot carcass weight and dressing percentage were observed with those supplemented with Nano-Se at all levels compared with other treatments. However, feed conversion, net revenue and economic efficiency values were improved with Nano-Se groups followed by organic Se group in comparisons with the control group. Conclusively, the Nano-Se in rabbit’s diet has a positive effect in improving rabbit^’^s performance and economic efficiency compared to the inorganic Selenium.

## Introduction

Rabbits are an excellent source of protein for human consumption so they can solve a part of the meat shortage in Egypt. Egypt has the fifth position in the production of rabbit meat (69.840 million tons) after China, Italy, Spain, and France (FAO 2004). Egypt's population is still developing intensively, so many investigations have been done to explore a new technology to improve feed efficiency and increase the growth performance of rabbits. Nanotechnology is considered a tool to explain the metabolic and physiological mechanisms of animal nutrition, and also a tool to improve fiber digestion in animals and to maintain its healthy status (Rajendran et al. 2014). Recently, using the Nano technology such as Nano minerals supplementation in diets indicated that nanoparticles of mineral elements had a higher bioavailability at low dosages compared with inorganic or organic mineral sources due to greater specific surface area, higher surface activity, high catalytic efficiency, and strong adsorbing ability (Chaudhry and Castle, 2011, Shi et al. 2011, Albanese et al. 2012 and Rajendran et al. 2014). Minerals bioavailability in the form of inorganic sources is quite low so these minerals are added 20–30 fold more than the normal requirement of animals resulting in environmental pollution by excess excretion of these minerals in feces (|Kanti et al. 2018). Selenium plays an important role in antioxidant defense mechanism, it prevents cell damage and it is necessary for growth, fertility, and immunity in farm animals (Rajendran et al. 2014; Michalak et al. 2022). The inorganic and organic forms of Se (selenite, selenide, selenium-enriched yeast, and selenium-enriched algae) are used as conventional supplements. Recently, many researchers reported that Nano-Se possessed a higher efficiency than selenite, selenomethionine, and methyl selenocysteine (Izabela Michalak et al. 2022). Using trace minerals in a nanoscale may be more effective for the productive and reproductive performance of the rabbits when used different sources of some trace minerals in the inorganic and organic forms of Se and Nano form (Fatma et al. 2016; Mohamed et al. 2016). Nano-Se supplementation had a positive impact on the growth performance, feed efficiency of the rumen and fertility (Rajendran et al. 2014) as well as promotes antioxidant activity, improves immune response, intestinal health, and nutritional value of animal products and low toxicity (Huang et al. 2015).

This study was undertaken to evaluate the effect of different forms of selenium elements (inorganic, organic, and Nano-Se) in rabbit’s diet on growth performance, nutrient digestibility, carcass traits, some blood plasma biochemical and histological features of liver, kidney, and economic efficiency of growing New Zealand White rabbits during the fattening period (6–14 weeks of age).

## Materials and methods

### Ethical approval

The present study was conducted at Sakha, Experimental Research Station, Kafr El-Sheikh Governorate, Animal Production Research Institute, Agriculture Research Center, Ministry of Agriculture, Dokki, Giza, Egypt and registered with a serial number (372,429).The study was respectfully carried out in manner of the stated ethics and animal rights (DRC) in accordance with the European Union Directive Regulations (2010/63/EU) regarding the protection of animals used for experimental and other scientific purpose.

#### Preparation

Selenium nanoparticles were prepared in the laboratory of the Poultry Nutrition Department, Animal Production Research Institute, Agricultural Research Center. Selenium nanoparticles were prepared by the reduction of selenium with diluted aqueous solutions containing Sodium sulfite (Na_2_SO_3_). Sodium selenosulphate solution was prepared by refluxing a mixture of selenium and Na_2_SO_3_ (Sigma company, Australia) in double distilled water at 70-80 °C for about 7-8 hours (Gorer and Hodes 1994). An aqueous polyvinyl alcohol (PVA) stock solution, 1% by weight, was prepared and used as stabilizing agent.

The formation of orange-red colored selenium nanoparticle solution was observed in less than one minute upon mixing the PVA with Sodium selenosulphate. It is important to use stabilizer, during the preparation of metal nano-particle, to avoid Nano-particles agglomeration (Bai et al. 2007).

#### Characterization

**Fig. 1:**
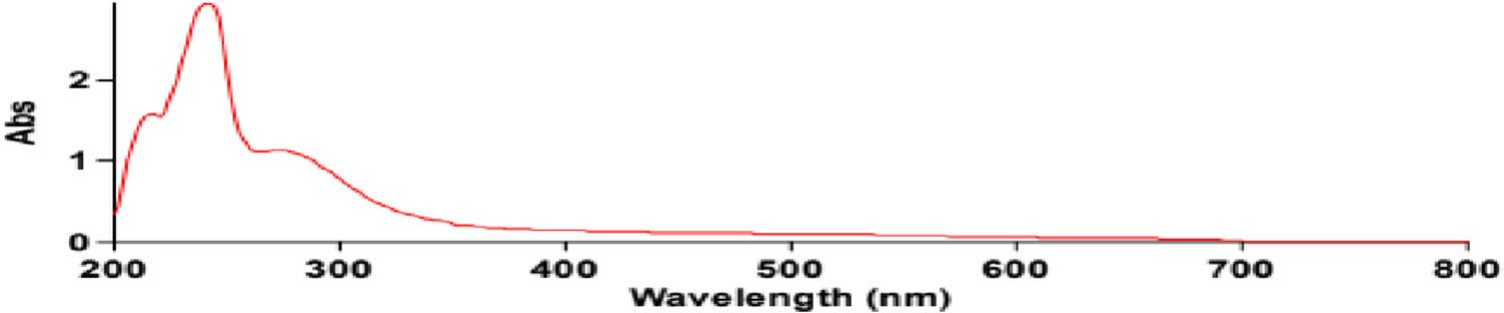
UV-V spectroscopy of Nano-Se

Characterization of nanoparticles is important to understand and control nanoparticles synthesis and applications. Finally, silver nanoparticles (Ag-NPs) gross was assessed by UV-V spectroscopy and the average of Particle size and the size distribution were determined from transmission electron microscopy (TEM) (Fig. 1). The determination of SeNPs concentration of the present study was 17,800 ppm per liter using Atomic absorption spectrometry (Agilent Technologies 200 Series AA), in the Egyptian National Research Center (NRC).

#### Animals and management

Forty-eight weaned male New Zealand White rabbits aged 6 weeks with an initial body weight of (709.67 ± 13.3 g) were randomly divided into six experimental groups (8 rabbit each). All rabbits were individually kept in battery cages (30 × 60 × 40 cm) within natural ventilated building with ambient temperature of 22–25 ^o^ C, relative humidity (60- 70%) and lighting regime of 14 h light and 10 h dark. The experimental period lasted for 14 weeks of age during November-February interval. All rabbits were kept under the same managerial, hygienic and environmental conditions on the farm.

#### Experimental diets

Six experimental treatments in completely randomized design were used. The first group (G_1_) as control was fed a basal diet containing 0.1 mg Na_2_SeO_3_/kg (sodium selenite) inorganic Se form to cover maintenance and production allowances according to NRC (1977). Rabbits in the other groups G_2_, G_3_, G_4_, and G_5_ were fed the basal diet (without inorganic selenium) supplemented with Nano Se at 20, 30, 40 and 50% of sodium selenite requirement, respectively (0.02, 0.03, 0.04 and 0.05 mg/kg diet, respectively). The 6^th^ group (G_6_) was fed the basal diet (without inorganic selenium) supplemented with 0.1 mg salinized yeast (Se-yeast) /kg diet as organic form. All rabbits were fed *ad- libtum* and fresh water was automatically available all times. The composition and calculated analysis of the basal diet are shown in Table 1.

**Table 1:** The composition and calculated analysis of the basal diet

Ingredient	%	Calculated chemical analysis	
Berseem hay	30.05	Crude protein (%)	17.75
Barley	24.60	Digestible energy kcal/kg	2500
Wheat bran	21.50	Crude fiber (%)	12.38
Soybean meal (44% CP)	17.50	Ether extract (%)	2.27
Molasses	3.00	Calcium (%)	1.24
Di-calcium phosphate	1.60	Total phosphorus (%)	0.80
Limestone	0.95	Lysine (%)	0.98
Sodium chloride (NaCl)	0.30	Methionine (%)	0.46
Vitamin & Mineral Mixture*	0.30	Methionine + Cystine (%)	0.76
DL-Methionine	0.20	Sodium (%)	0.16

*Supplied per kilogram of diet: Vitamin A, 6000 IU; Vitamin D_3_, 900 IU; Vitamin E, 40 mg; Vitamin K_3_, 2 mg; Vitamin B_1_, 2 mg; Vitamin B_2_, 4 mg; Vitamin B_6_, 2 mg; Pantothenic acid, 10 mg; Vitamin B_12_, 0.01 mg; Niacin, 50 mg; Folic acid, 3 mg; Biotin, 0.05 mg; Choline, 250 mg; Fe, 50 mg; Mn, 8.5 mg; Cu, 5 mg; Co, 0.1 mg; Se, 0.1 mg; I, 0.2 mg and Zn, 50 mg

#### Digestibility trials

At the end of the experimental period, a digestibility trial was conducted to determine the digestibility coefficient of the nutrients. Six digestion experiments were conducted by using three rabbits from each group randomly at the age of 14 week. Rabbits were housed individually in metabolically galvanized metal cages (60 × 50 × 40 cm) ) that allowed separating feces from urine and supplied with separated feeders for ten days as preliminary and five days as the collection period. Feces were collected separately every 24 hours and sprayed with a 2% boric acid solution to trap ammonia released from feces. At the end of this period, feces were dried at 60° c for 48 h (till constant weight), finely ground, band thoroughly mixed to ensure sample uniformity, and then stored until being analyzed according to A.O.A.C. (1995).

#### Growth performance

Live body weight (LBW); weight gain (final weight– initial weight) and feed intake were weekly recorded, and feed conversion (daily feed intake / daily gain,) ratio was calculated, all the different parameters were calculated based on gram (g).

#### Carcass traits

At the end of experimental period, three rabbit were randomly taken from each treatment and fasted for16 hours before slaughtering to determine the carcass characteristics (carcass, edible giblets, liver, heart, kidney, spleen, total non-carcass fat%, Fur and dressing carcass %) according to Hassan et al. (2016).

#### Blood sampling

During slaughtering, blood samples (5 ml/each sample) were taken individually from three rabbits in each group (18 samples). Blood samples were collected in dry clean centrifuge tubes containing few drops of heparin solution and centrifuged at 3000 rpm. for 20 minutes to separate blood plasma and stored in deep freezer at approximately -20°C until the time of analysis to estimate blood parameters Abdulla et al. (2019), Kairalla et al. 2022) . Blood plasmas simples were determined by calorimetry spectrophotometer (Spectronic 21 DUSA) using commercial diagnostic kits supplied by Bio Merieux, France, following the same steps described by manufactures. The blood plasma was measured for total protein according to Gornall et al. (1949), albumin according to Doumas et al. (1971), while plasma globulin value was determined as plasma total protein minus that of albumin. Glucose concentration was evaluated according to Coles (1986). Kidney function (urea and creatinine) was assessed by measuring urea -N according to Fawcett and Scott (1960) and creatinine concentration according to Schirmeister et al. (1964). The liver function (AST and ALT) were measured according to method of Reitman and Frankel (1957). Fat fractions (cholesterol, tri-acyl glycerol, HDL and LDL) were measured according to Richmond (1973) for total cholesterol and triglycerides according to Fassati and Prenciple (1982) HDL concentration by Warnick et al. (1983) and LDL by Friedwald et al. (1972). The thyroid hormones (T_3_ and T_4_) were determined by Tietz 1995, in immunoassay.

#### Histological study

Representative samples were taken from the median part of liver and kidney of slaughtered male rabbits from each group, put in normal formalin (10 % solution, 38-40%) at 24- 48 hours, then washed by tap water for 24 h and dehydrated in ascending grades of ethyl alcohol (50-100 %), cleared, routinely processed and sectioned by microtome at 5-7 microns, thickness. Sections were mounted on glass slides, deparaffinized with xylol and stained by hematoxylin and eosin (H&E) stains and histologically examined using a light microscope (Optico XSZ-107B Binocular, China) at x200 magnifications.

#### Economic efficiency

Economic efficiency was calculated as the ratio between incomes price of daily weight gain and cost of feed consumed at 14 weeks of age.

$$\begin{array}{l}{\mathrm{Net\; revenue }\left({\text{LE}}\right)}^{*}=\mathrm{ Total \;revenue}-\mathrm{Total\; feed \;cost}.\\ \mathrm{Economic\; efficiency\; \% }=\mathrm{ Net\; revenue }({\text{LE}})/\mathrm{ Total\; feed\; cost }\;({\text{LE}}).\end{array}$$

The price of ingredients and selling of one kg of live weight of rabbits was calculated according to the price in the local market at the time of experiment during January 2020.The price of one kg live body weight was 60 Egyptian Pound (LE).

#### Statistical analysis

Data were subjected to statistical analysis as a one-way classification analysis of variance (Completely Randomized Design) using the general liner models procedure of SAS (1997) according to the following fixed model:

$${{\text{Y}}}_{{\text{ij}}}=\upmu + {{\text{T}}}_{{\text{i}}}+ {{\text{e}}}_{{\text{ij}}}$$

where: Y_ij_ = the observation, µ= overall mean, T_i_ = effect of treatments (i: 1 to 6.), e_ij_ = random error component assumed to normally distributed. Duncan's multiple range tests was performed (Duncan 1955) to detect significant differences among means values between treatments.

## Results

### Nutrients digestibility and nutritive value

Data in Table 2 showed that the rabbit fed the control diet (G_1_) had the lowest values in all digestibility (%) and nutritive values (%) compared with those fed diets supplemented with Nano-Se levels or organic -Se. However the NFE and CF digestibility were significantly (*p* ≤ 0.05) higher with rabbits fed a diet containing both Nano and organic-se than that in control diet (G_1_). Nano-Se supplementation in rabbit diets (G_4_) was slightly increased all nutrient digestibility values (%) and nutritive value (%) compared with other levels of Nano-Se.

**Table 2:** Nutrients digestibility and nutritive values of rabbit’s diet supplemented with different selenium forms

	Experimental groups					
Items	G_1_ (control, inorganic Se)	Nano-Se				G_6_ (Se-yeast)
		G_2_	G_3_	G_4_	G_5_	
Digestibility, %						
DM	63.84 ± 1.68	70.74 ± 3.17	73.01 ± 2.20	73.08 ± 2.84	71.04 ± 1.93	71.44 ± 3.58
OM	69.97 ± 1.94	75.10 ± 3.74	75.18 ± 3.79	77.18 ± 2.24	75.65 ± 1.54	74.86 ± 3.54
CP	75.65 ± 1.30	77.21 ± 3.39	77.24 ± 3.73	77.46 ± 2.00	76.35 ± 1.58	76.28 ± 3.28
EE	81.45 ± 5.18	81.87 ± 5.80	82.58 ± 5.50	84.58 ± 6.84	82.55 ± 5.49	82.22 ± 5.90
CF	36.52 ^b^ ± 1.40	46.97^a^ ± 2.11	48.07 ^a^ ± 1.62	48.38 ^a^ ± 1.52	45.48 ^a^ ± 2.60	44.89 ^a^ ± 2.70
NFE	77.39 ^b^ ± 1.41	83.15 ^a^ ± 2.77	84.52 ^a^ ± 3.43	84.56 ^a^ ± 2.3	84.42 ^a^ ± 1.30	82.77 ^a^ ± 3.50
Nutritive values, %						
TDN	65.97 ± 1.14	71.76 ± 2.70	72.83 ± 2.00	72.82 ± 2.38	71.66 ± 1.40	71.83 ± 3.00
DCP	10.74 ± 0.29	11.23 ± 0.43	11.43 ± 0.53	11.73 ± 0.13	11.71 ± 0.45	11.60 ± 0.53

^a,b^ means in the same row with different superscripts are significantly different (*P* ≤ 0.05)

G_1_: control contained 0.1 mg sodium-Se /kg of diet

G_2_: contained 0.02 mg/kg Nano-Se of diet

G_3_: contained 0.03 mg/kg Nano-Se of diet

G_4_: contained 0.04 mg/kg Nano-Se of diet

G_5_: contained 0.05 mg/kg Nano-Se of diet

G_6_: 0.1 mg Se-yeast /kg of diets

*DM* Dry matter, *OM* Organic matter, *CP* Crude protein, *EE* Ether extract, *CF* Crude fiber, *NFE* Nitrogen free extracts, *TDN* Total digestible nutrients, *DCP* Digestible crude protein

### Growth performance

Data in Table 3 showed that average live body weight (LBW) in dietary rabbit supplemented with Nano-Se (G_2_, G_3_, G_4_ and G_5_) and organic-Se (G_6_) where significantly higher (*p* ≤ 0.05) compared with the control group (G_1_) at 14 weeks. While the best average LBW was recorded with G_5_ during the same period. Average daily gain (g/head) was increased (*p* ≤ 0.05) with Nano-Se supplementation at all levels and organic form (G_6_) in comparison with those in control group (G_1_) at 6–14 weeks. Data in Table 3 showed that dietary rabbits supplemented by Nano-Se or organic Se had no significant effect on total feed intake (g/head/week) and average daily feed intake (g/head/day) compared with the control group (G_1_) at all studied periods. However, feed conversion ratio was significantly (*p* ≤ 0.05) improved in all treatments compared to the control group (Table 3). On the other hand, the best feed conversion ratio (gm, feed intake / gm, daily weight gain) was found with those fed diets supplemented by Nano-Se (G_5_) compared with other treatments at all studied periods.

**Table 3:** Growth performance values of growing male rabbits as affected by selenium forms supplementation at different ages

	Experimental groups					
Items	G_1_(control, inorganic Se)	Nano-Se				G_6_ (Se-yeast)
		G_2_	G_3_	G_4_	G_5_	
Live Body Weight, g						
6 WK (Initial Weight)	718.0 ± 18.03	714.0 ± 15.77	711.0 ± 15.36	698.0 ± 17.76	697.0 ± 11.38	720.0 ± 17.03
10 WK	1306.0 ^b^ ± 25.98	1408.0^abc^ ± 26.50	1435.0 ^ab^ ± 25.73	1454.4 ^a^ ± 23.91	1441.0 ^a^ ± 25.72	1444.0 ^a^ ± 26.99
14 WK	1925.5 ^b^ ± 28.03	2128.0 ^a^ ± 23.45	2141.1 ^a^ ± 29.21	2145.0 ^a^ ± 30.17	2183.7 ^a^ ± 30.32	2161.8 ^a^ ± 31.06
Daily weight gain (g/head)						
(6 -10) weeks	21.00 ^c^ ± 0.87	24.78 ^ab^ ± 1.05	25.86 ^a^ ± 1.53	27.01 ^a^ ± 1.61	26.57 ^a^ ± 0.88	25.86 ^a^ ± 1.02
(10–14) weeks	22.13 ^c^ ± 1.08	25.71 ^a^ ± 1.56	25.22 ^a^ ± 1.52	24.66 ^b^ ± 1.25	26.53 ^a^ ± 0.59	25.64 ^a^ ± 1.08
(6–14) weeks	21.55 ^b^ ± 0.46	25.25 ^a^ ± 0.45	25.54 ^a^ ± 0.34	25.84 ^a^ ± 0.40	26.56 ^a^ ± 0.52	25.75 ^a^ ± 0.47
Total feed intake, g/head/week						
at (6 -10) weeks	434.8 ± 6.17	422.1 ± 7.89	430.4 ± 6.69	442.6 ± 6.35	445.3 ± 5.70	428.9 ± 9.34
at (10–14) weeks	722 ± 10.98	740 ± 11.41	737.3 ± 11.54	736.9 ± 10.53	757.4 ± 11.27	753 ± 10.45
at (6–14) weeks	578.4 ± 8.57	581.1 ± 9.65	583.9 ± 9.12	589.8 ± 8.44	601.4 ± 8.48	591 ± 9.90
Daily feed intake, g/head/d						
FI (6–10)	62.11 ± 0.74	60.30 ± 0.85	61.49 ± 0.81	63.23 ± 0.76	63.61 ± 0.79	61.27 ± 0.81
FI (10–14)	103.14 ± 0.78	105.71 ± 0.98	105.33 ± 1.19	105.27 ± 1.01	108.20 ± 0.62	107.57 ± 0.96
FI (6–14)	82.63 ± 0.42	83.01 ± 0.66	83.41 ± 0.74	84.25 ± 0.55	85.91 ± 0.34	84.42 ± 0.71
Feed conversion ratio, g, feed intake/g, daily weight gain						
(6–10) weeks	2.96 ^a^ ± 0.10	2.43 ^b^ ± 0.08	2.38 ^b^ ± 0.14	2.34 ^b^ ± 0.13	2.39 ^b^ ± 0.09	2.37 ^b^ ± 0.11
(10 -14) weeks	4.66 ^a^ ± 0.21	4.11 ^ab^ ± 0.25	4.18 ^b^ ± 0.18	4.27 ^b^ ± 0.16	4.08 ^**b**^ ± 0.15	4.20 ^**b**^ ± 0.11
(6–14) weeks	3.83 ^a^ ± 0.07	3.29 ^b^ ± 0.12	3.27 ^b^ ± 0.13	3.26 ^b^ ± 0.11	3.23 ^b^ ± 0.05	3.29 ^**b**^ ± 0.04

^a,^ ^b,^ ^c^means in the same row with different superscripts are significantly different (*P* ≤ 0.05)

G_1_: control contained 0.1 mg sodium-Se /kg of diet

G_2_: contained 0.02 mg/kg Nano-Se of diet

G_3_: contained 0.03 mg/kg Nano-Se of diet

G_4_: contained 0.04 mg/kg Nano-Se of diet

G_5_: contained 0.0 5 mg/kg Nano-Se of diet

G_6_: 0.1 mg Se-yeast /kg of diets

### Carcass traits

Data of carcass traits (Table 4) showed that, the hot carcass weight% and dressing percentage were significantly (*p* ≤ 0.05) increased in all treatments (Nano or organic-Se) compared to the control group. The highest values of carcass weight percentage were increased with increasing the levels of Nano-Se, and the highest one occurred in the G_5_ group. Relative weights % of kidney, liver, heart, spleen, giblets, and fur were numerically similar among experimental groups. On the other hand, the relative weight % of the head and total non-carcass fat were the lowest values (*p* ≤ 0.05) within the control group compared with the other treatments.

**Table 4:** Carcass traits of growing male rabbits fed rations supplemented with different forms of selenium

	Experimental groups					
Items	G_1_ (control, inorganic Se)	Nano-Se				G_6_ (Se-yeast)
		G_2_	G_3_	G_4_	G_5_	
Preslaughter weight(g)	1930	1946.66	2105.00	2113.33	2130.00	2153.30
Hot carcass weight %	42 ^d^ ± 1.22	45.96^ cd^ ± 1.22	47.58^bc^ ± 1.04	51.48 ^ab^ ± 1.19	52.42 ^a^ ± 1.33	50.58 ^ab^ ± 1.76
Dressing %	54.77 ^b^ ± 2.21	60.04 ^a^ ± 1.06	61.15 ^a^ ± 0.29	62.08 ^a^ ± 0.33	62.36 ^a^ ± 0.67	61.85 ^a^ ± 0.73
Liver%	2.52 ± 0.11	2.53 ± 0.25	2.55 ± 0.52	2.48 ± 0.09	2.68 ± 0.21	2.54 ± 0.16
Kidneys, %	0.84 ± 0.05	0.79 ± 0.06	0.82 ± 0.05	0.84 ± 0.03	0.78 ± 0.01	0.81 ± 0.05
Heart, %	0.31 ± 0.05	0.32 ± 0.06	0.33 ± 0.04	0.35 ± 0.04	0.34 ± 0.01	0.33 ± 0.01
Spleen, %	0.06 ± 0.005	0.06 ± 0.011	0.04 ± 0.002	0.05 ± 0.009	0.04 ± 0.005	0.05 ± 0.01
Edible giblets, %	3.67 ± 0.01	3.64 ± 0.68	3.70 ± 0.80	3.67 ± 0.06	3.80 ± 0.51	3.68 ± 0.40
Total Non-carcass fat%	0.48 ^b^ ± 0.10	1.38 ^ab^ ± 0.42	1.22 ^ab^ ± 0.36	2.21 ^a^ ± 0.18	2.12 ^a^ ± 0.29	1.82 ^ab^ ± 0.18
Head, %	4.55 ^b^ ± 0.24	5.76 ^ab^ ± 0.31	5.69 ^ab^ ± 0.31	6.26 ^a^ ± 0.27	5.84 ^ab^ ± 0.25	5.57 ^ab^ ± 0.24
Fur, %	20.90 ± 0.64	20.85 ± 1.33	18.10 ± 0.65	17.78 ± 0.31	18.64 ± 0.33	18.71 ± 0.48

^a,b,c,d^ means in the same row with different superscripts are significantly different (*P* ≤ 0.05)

G_1_: control contained 0.1 mg sodium-Se /kg of diet.

G_2_: contained 0.02 mg/kg Nano-Se of diet.

G_3_: contained 0.03 mg/kg Nano-Se of diet.

G_4_: contained 0.04 mg/kg Nano-Se of diet.

G_5_: contained 0.05 mg/kg Nano-Se of diet.

G_6_: 0.1 mg Se-yeast /kg of diets

### Blood plasma and histological study

**Fig. 2:**
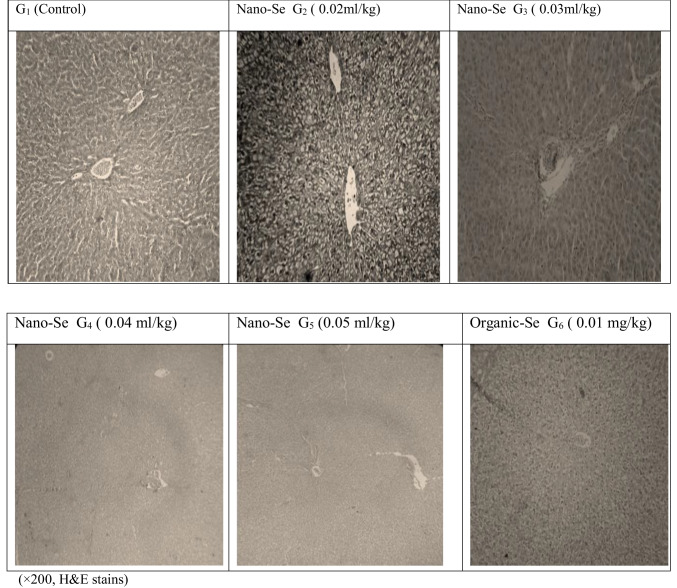
The histological examination of liver of slaughtered rabbits

**Fig. 3:**
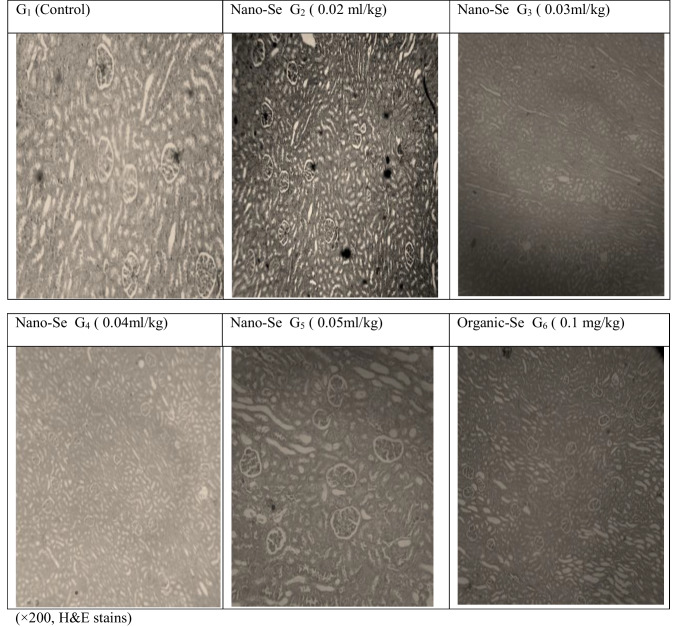
The histological examination of Kidneys of slaughtered rabbits

The results in Table 5 showed that dietary rabbits supplemented with different forms of selenium had no significant effect on all blood plasma parameters, except for the T_4_ was significantly (*p* ≤ 0.05) higher for both G_5_ and G_4_ (Nano-Se), followed by G_6_, G_3_ and G_2_, and finally G_1_ (control group). On the other hand, the histological examination of the liver of slaughtered rabbits (Fig. 2) showed that there is the typical architecture of the hepatic lobules in all treated groups (G_2_, G_3_, G_4_, G_5_ and G_6_) as in the control group. The histological examination of the kidneys of slaughtered rabbits (Fig. 3) showed the typical architecture of the renal cortex and medulla in all groups.

**Table 5:** Blood plasma parameters of male rabbits as influenced by different selenium forms supplementation

	Experimental groups					
Items	G_1_ (control, inorganic Se)	Nano-Se				G_6_ (Se-yeast)
		G_2_	G_3_	G_4_	G_5_	
Total protein (g/dl)	6.73 ± 0.23	6.97 ± 0.23	7.10 ± 0.30	6.50 ± 0.20	7.20 ± 0.06	7.13 ± 0.33
Albumin (g/dl)	3.94 ± 0.22	4.04 ± 0.17	3.94 ± 0.11	3.78 ± 0.23	4.17 ± 0.10	4.16 ± 0.32
Globulin (g/dl)	2.79 ± 0.28	2.93 ± 0.22	3.16 ± 0.19	2.72 ± 0.25	3.03 ± 0.11	2.98 ± 0.02
Albumin/globulin	1.45 ± 0.08	1.39 ± 0.12	1.25 ± 0.06	1.43 ± 0. 10	1.38 ± 0.08	1.40 ± 0.10
Cholesterol (mg/dl)	122.67 ± 11.61	112.33 ± 13.17	125.00 ± 14.01	120.33 ± 12.01	124.67 ± 13.67	129.67 ± 12.55
HDL (mg/dl)	54.90 ± 5.88	58.87 ± 5.72	55.73 ± 4.06	56.80 ± 5.36	57.43 ± 6.66	54.93 ± 6.27
LDL (mg/dl)	43.90 ± 5.62	44.13 ± 6.33	50.40 ± 6.07	41.73 ± 6.96	36.33 ± 4.03	54.27 ± 4.87
Tri-acyl glycerol (mg/dl)	119.33 ± 8.18	95.33 ± 7.33	94.33 ± 4.91	109.00 ± 9.16	103.33 ± 6.66	102.33 ± 9.24
Glucose (mg/dl)	68.00 ± 3.27	65.33 ± 4.05	65.33 ± 3.66	69.33 ± 3.61	66.00 ± 4.14	63.33 ± 3.40
Urea (mg/dl)	34.67 ± 3.24	40.33 ± 3.38	40.33 ± 3.84	39.33 ± 2.88	40.33 ± 3.33	43.00 ± 3.21
Creatinine (mg/dl)	1.46 ± 0.199	1.46 ± 0.137	1.56 ± 0.102	1.51 ± 0.029	1.50 ± 0.141	1.61 ± 0.058
AST (U/l)	41.67 ± 5.89	50.67 ± 6.35	53.33 ± 4.48	51.33 ± 5.60	50.00 ± 6.03	50.33 ± 6.94
ALT (U/l)	64.00 ± 11.53	67.33 ± 7.05	71.00 ± 12.76	52.67 ± 6.56	65.00 ± 12.01	66.33 ± 16.25
T_3_ (ng/ml)	1.28 ± 0.08	1.00 ± 0.04	1.03 ± 0.04	1.09 ± 0.08	1.04 ± 0.07	1.25 ± 0.23
T_4_ (ng/ml)	2.38 ^b^ ± 0.57	2.63 ^ab^ ± 0.19	2.68 ^ab^ ± 0.22	2.81 ^a^ ± 0.20	2.83 ^a^ ± 0.23	2.75 ^ab^ ± 0.32

^a, b^ means in the same row with different superscripts are significantly different (*P* ≤ 0.05)

G_1_: control contained 0.1 mg sodium-Se /kg of diet

G_2_: contained 0.02 mg/kg Nano-Se of diet

G_3_: contained 0.03 mg/kg Nano-Se of diet

G_4_: contained 0.04 mg/kg Nano-Se of diet

G_5_: contained 0.05 mg/kg Nano-Se of diet

G_6_: 0.1 mg Se-yeast /kg of diets

*HDL* High-density lipoprotein, *LDL* Low-density lipoprotein, *AST* Aspartate transaminase, *ALT* Alanine transaminase, *T*_*3*_ Triiodothyronine, *T*_*4*_ Thyroxine

### Economic efficiency

From the economic point of view (Table 6), it could be indicated that feeding growing rabbits from 6–14 weeks of age on diets supplemented with Nano-Se raised the net revenue and economic efficiency values compared with those fed diets supplemented with either organic-Se or a control group which contained inorganic -Se. Increasing levels of Nano-Se particles resulted in increased the economic efficiency values compared with the organic-Se or the control group. Whereas, rabbit diets supplemented with organic-Se (G_6_) had a higher value than that of control group (G_1_). The best relative economic efficiency was found with the G_5_ group (137.5%, Nano-Se particles) than those in other groups.

**Table 6:** Economical efficiency of experimental diets supplemented with different selenium forms

	Experimental groups					
Items	G_1_ (control, inorganic Se)	Nano-Se				G_6_ (Se-yeast)
		G_2_	G_3_	G_4_	G_5_	
Total body weight gain (kg)	1.208	1.414	1.430	1.447	1.487	1.412
Price of 1 kg body weight (l.E)	60	60	60	60	60	60
Selling price/rabbit (L.E) A	72.48	84.84	85.80	86.82	89.22	84.72
Total feed intake (kg)	4.627	4.648	4.671	4.718	4.811	4.729
Price of 1 kg feed (L.E.)	8	8	8	8	8	8
Total feed cost/rabbit (L.E) B	37.02	37.18	37.37	37.74	38.49	37.83
Net revenue (L.E)^1^	35.46	47.66	48.43	49.08	50.73	46.89
Economic efficiency ^2^	0.96	1.28	1.30	1.30	1.32	1.24
Relative EEF, %	100	133.3	135.4	135.4	137.5	129.2

^1^Net revenue = A-B, ^2^ Economical efficiency = Net revenue / B

G_1_: control contained 0.1 mg sodium-Se /kg of diet

G_2_: contained 0.02 mg/kg Nano-Se of diet

G_3_: contained 0.03 mg/kg Nano-Se of diet

G_4_: contained 0.04 mg/kg Nano-Se of diet

G_5_: contained 0.05 mg/kg Nano-Se of diet

G_6_: 0.1 mg Se-yeast /kg of diets

## Discussion

### Nutrients Digestibility and nutritive value

The present research was designed to evaluate the effect of using different forms of Se element (inorganic, nanoparticles, and organic) as dietary supplementation on the productive performance of New Zealand White Rabbits. The Nano-Se supplementation at all levels recorded the highest values for DM, OM, CP, CF and NFE digestibility (%) and nutritive values (%) than those other treatments.

This may be due to Nano-Se additives, which are characterized by the largest specified surface area, high surface activity, high motivational efficiency, and strong absorption capacity, although additives them in simple doses. Similar results were obtained by Chaudhry and Castle (2011), Shi et al. (2011), Albanese et al. (2012), Rajendran et al. (2014) who reported that Nano particles were higher bioavailability at low doses compared with inorganic or organic mineral sources. Also, Rajendran et al. (2014) recorded that dietary Nano-Se supplementation could be a higher efficiency than those in selenite, selenomethionine, and methyl selenocysteine in upregulating selenoenzymes in mice and rats. Data in Table 2 showed that all levels of the Nano-Se supplementation in the rabbits' diet improved CF digestibility by 28.8, 31.63, 32.48, and 25.23% for groups G_2_, G_3_, G_4_, and G_5_, respectively. On the other hand, the animals fed G_6_ improved CF digestibility by 22.92% compared to those fed the control rations (G_1_). These results in agreement by Thulasi et al. (2014) who found that nanotechnology improved fiber digestion and feed utilization in ruminant. While, Amer et al. (2019) reported that crude fiber digestibility of growing male New Zealand White rabbits significantly increased by 28% when fed the diet supplemented with organic selenium in comparison with those fed the control diet.

### Growth performance

The current results revealed that the average live body weight (LBW) and daily gain were higher in dietary rabbits supplemented by different levels of Nano-Se compared with the control group. This effect could be considered a response to increased bioavailability, specific surface area, surface activity, catalytic efficiency, and strong adsorbing ability at the different levels of Nano-Se. This led to an increase in all digestibility (%) and nutritive values (%), which was reflected in increase average LBW and daily gain performance. These results are in agreement with Abdel-wareth et al. (2019), Emara et al. (2019), Sheiha et al. (2020) who recorded that dietary supplementation of Nano-Se revealed heaviest than selenium selenite and control without supplemented Se, due to better absorption and higher bioavailability of Nano-Se. These results were agreed with Emara et al. (2019) for growing New Zealand rabbits and Diana et al. (2020), for female New Zealand rabbits at 8 weeks old, who reported that daily gain of rabbet increased by the Nano-Se additives with the diets. On the other hand, the total feed intake and average daily feed intake were not affected by Nano-Se or organic Se addition compared with the control ration. In contrast with our results, Zeweil et al. (2016) reported that adding different sources of Se to rabbit diets significantly reduced feed intake and feed conversion in different treatments compared to the control. On the other hand, EL-Deep et al. (2017) indicated that the addition of Nano-Se (0.3 mg/kg) into the Cockerels Inshas (Egyptian local strain at 42-week old under heat stress conditions in the summer season) diet did not affect the feed intake during high environmental temperatures.

However, the feed conversion ratio was considered an important factor in measuring the productive performance of rabbits. It was found that the feed conversion ratio improved in all treatments (Nano or organic-Se) compared with those in the control rations. This may be due to the Nano-Se additions improved all digestibility (%) and nutritional values (%), and then an increase in average body weight and daily gain performance, which was reflected in an improvement in the feed conversion ratio in the animals fed diets supplemented Nano-Se compared with the control rations. These results are agreement with those obtained by Ebeid et al. (2012) for organic-Se and Abdel-Wareth et al. (2019) for Nano-Se who reported that the Nano or organic-Se were significantly improved feed conversion when compared to the control group. On another note, the improvement in growth performance by Nano-Se supplementation may be due to its higher bioavailability of Nano-Se, better absorption, high oxidation resistance and high immune regulating (Huang et al. 2015; Emara et al. 2019).

### Carcass traits

The current results showed that rabbits fed Nano or organic -Se as feed additives improved the hot carcass weight and dressing percentage compared with the control group. This is due to an increase in both live body weight and average daily gain, which was reflected in an improvement in the different carcass traits. This present result is comparable to that obtained by Sheiha et al. (2020) who reported that supplemented with Nano-Se synthesized by biological method (BIO25 and BIO50, with a 25 and 50 mg of nano-Se/kg diet, respectively) on growing rabbit's diet at 50 mg/kg significantly increased growth performance and carcass criteria of rabbits compared to control. Also, Ebeid et al. (2012) found that supplemented diets with organic Se (0.15 or 0.30 ppm) improved the carcass weight and dressing percentage of male California rabbits compared with the control. In contrast with our results, Noha (2019), Dokoupilová et al. (2007) reported that Nano-Se supplementation for growing rabbit’s diet did not affect the dressing percentage and total edible parts weight. However, studies reported by Selim et al. (2015) pointed out a Se-Yeast or Se-Methionine as the organic form of selenium or L-Nano Se as the Nano form of Se at a level of 0.30 ppm in broiler diets or its equivalent in drinking water are more effective to get better growth performance and quality of broiler meat.

### Blood plasma parameters and histological study

The current results showed that dietary rabbits supplemented with different forms of selenium had no significant effect on all blood plasma parameters, except for T_4_ which was significantly (*p* ≤ 0.05) higher for both G_5_ and G_4_ (Nano-Se), followed by G_6_, G_3_, and G_2_, and finally G_1_ (control group). This indicates the remarkable adaptive abilities of growing rabbits to feed at different levels of Nano-Se without damaging the liver and kidneys, which was reflected in the productive performance of growing rabbits. In the histological study, it was noted that using Nano-Se as feed additives for rabbits improved digestibility, nutritional values, feed conversion ratio, and daily growth. Histological examination of the liver and kidneys of the slaughtered rabbits showed that there was a normal structure of the hepatic lobule and renal cortex in all treated groups. This is due to the blood parameters remained within normal physiological ranges. This indicates the remarkable adaptive abilities of developing rabbits to deliver high levels of productive performance without compromising liver and kidney function. This present result is comparable to that obtained by Selim et al. (2015) reported that plasma total proteins, albumin, globulin, albumin/globulins ratio and creatinine concentration were not significantly affected by Nano-Se supplementation to broiler diet. Also, Qin et al. (2016) found that blood total protein, cholesterol, HDL, triglyceride, ALT and AST concentrations were not significantly affected by Nano-Se or sodium selenite supplementation in growing rabbits. Plasma biochemical parameters, in this study were within the normal range of healthy rabbits according to (Alessandro Melillo, 2007).

### Economic efficiency

From the economic point, it could be concluded that feeding growing rabbit on diets supplemented with Nano-Se raised the net revenue and economic efficiency values compared with other treatments. Also, increasing levels of Nano-Se particles increased the economic efficiency values compared with the organic-Se or the control group. These results are in agreement with those reported by Hu et al. (2012) who found that the Nano-Se was more economical than the control group.

Generally, supplementing the animal diets with feed additives is considered to be important. In the present study, therefore using the Nano-Se as feed additives for the rabbit’s diet improved digestibility, nutritive values, feed conversion ratio, and daily growth rate and did not have a negative impact on the blood parameters and histological study (liver or kidney tissues) and the highest economic efficiency. More research attention is needed in this field to cater the benefit of these technologies in animal especially in nutritional science.

## Conclusion

Through to the present study, it can be concluded that adding Nano-Se at all levels, especially the G_5_ (Nano-Se 0.05 mg/kg), followed by organic selenium supplements in the rabbit's diet has been beneficial in improving the production performance of rabbits, which reflect on economic efficiency compared with the control group that contains inorganic-Se. In future studies, we need more research to explore the optimal safety levels of Nano Se supplementation for increasing productive and reproductive performance not only in rabbits but also with other classes of livestock without causing toxicity or hyper accumulation in animal tissues.

## Data Availability

The datasets generated during the current study are not publicly available because the research has not been published yet but are available from the corresponding author on reasonable request.
